# Providers’ perspectives on denial of abortion care in Nepal: a cross sectional study

**DOI:** 10.1186/s12978-018-0619-z

**Published:** 2018-10-11

**Authors:** Mahesh C Puri, Sarah Raifman, Biddhya Khanal, Dev Chandra Maharjan, Diana Greene Foster

**Affiliations:** 1 Center for Research on Environment, Health and Population Activities (CREHPA), Kathmandu, Nepal; 2 Advancing New Standards in Reproductive Health, University of California, San Francisco, USA

**Keywords:** Abortion, Providers, Denial of care, Quality of care, Referrals, Provider training, Nepal

## Abstract

**Background:**

Despite legalization of abortion in Nepal in 2002, many women are still unable to access legal services. This paper examines providers’ views, experiences with abortion denial, and knowledge related to abortion provision, and identifies areas for improvement in quality of care.

**Methods:**

We conducted a structured survey with 106 abortion care providers at 55 government-approved safe abortion facilities across five districts of Nepal in 2017. We assessed reasons for denial of abortion care, knowledge about laws, barriers to provision and attitudes towards abortion.

**Results:**

Almost all providers (96%) reported that they have ever refused clients for abortion services. Common reasons included beyond 12 weeks gestation (93%), sex selective abortion (86%), and medical contraindications (85%). One in four providers denied abortion for lack of drugs or trained personnel, and one third denied services when they perceived that the woman’s reasons for abortion were insufficient. Only a third of providers knew all three legal indications for abortion -- less than or equal to 12 weeks of pregnancy on request, up to 18 weeks for rape or incest, and any time for maternal or fetal health risk. Overall, providers were in favor of legal abortion but a substantial proportion had mixed or negative attitudes about the service.

**Conclusions:**

Improvements in training to address providers’ inadequate knowledge about the abortion law may reduce inappropriate denial of abortion. Establishing referral networks in the case of abortion denial and ensuring regular supply of medical abortion drugs would help more women access abortion care in Nepal.

## Plain English summary

Despite the legalization of abortion in 2002, many women in Nepal are still unable to access legal services. Past studies focused on women’s experiences being denied care. In this paper, we examine providers’ perspectives on abortion denial, reasons for denial of abortion, knowledge about abortion laws and safety, and attitudes about abortion. We surveyed 106 abortion providers in five districts and found that almost all providers (96%) had ever refused to provide abortion care. Providers reported the most common reasons for denying care were gestational age beyond 12 weeks, requests for a sex selective abortion, and/or medical conditions that would risk safety. Some providers were not adequately equipped with medications or trained staff, and others reported that the patient’s reasons for wanting an abortion were insufficient. Only one third of the providers were aware of all three stipulations of the abortion law in Nepal: 1) a woman may request an abortion up to 12 weeks in pregnancy for any reason, 2) a woman may request an abortion up to 18 weeks in pregnancy for reasons of rape or incest, and 3) a woman may obtain abortion any time in pregnancy for mental or physical health or risks to the fetus. High rates of abortion denial may be attributable to a lack of provider knowledge of the country’s abortion law, and providers may benefit from improved training. Established referral networks and consistent access to a supply of medications for abortion may also help improve access to services.

## Background

Since abortion was legalized in 2002, the Nepal government has taken important steps to include abortion as a component of reproductive health care, enabling many women to obtain safe, legal services. Under the law, women can request abortion up to 12 weeks gestation for any reason, up to 18 weeks for rape or incest, and with physician approval at any stage of pregnancy to protect mental or physical health and in cases of fetal anomaly [1]. Any pregnant woman with at least three out of 11 negative mental health conditions is eligible to receive an abortion after 12 weeks of gestation age [2]. Sex-selective abortion is prohibited and adult consent is required for girls under 16 years. The number of certified abortion clinics in Nepal has steadily expanded since 2004; by 2017, over 2000 clinicians and 532 facilities were trained and certified [3, 4]. Since 2008, nurses, in addition to physicians, have been eligible to receive training in manual vacuum aspiration (MVA) up to 8 weeks gestation. Second trimester abortion training for physicians began in 2007 and by 2017, 24 hospitals are providing second trimester abortion in the country. Forty-six providers had been trained and over 1800 women had been served [5]. In 2009, medical abortion was introduced (available within 9 weeks of gestation) and by 2017, primary health care centers and health posts located in 45 districts (out of 75) were providing medical abortion services.

However, research has shown that many Nepali women are still unable to access abortion services, especially the poorest, most disadvantaged and geographically isolated women [6]. An estimated quarter (26%) of Nepali women seeking abortion are denied the services [7]. An estimated 323,200 abortions were performed in Nepal in 2014, only 42% of which were provided legally in government-approved facilities [8]. Women may be denied abortion services due to providers’ lack of full understanding of the scope of the abortion law in the country. Though studies about women’s knowledge, experiences and perceptions of abortion service have been conducted in Nepal, abortion service providers’ knowledge, experiences and attitudes in providing abortion services are not fully known [6, 9]. To address this information gap, this paper presents findings from a study conducted with abortion care providers in Nepal.

## Methods

**Fig. 1:**
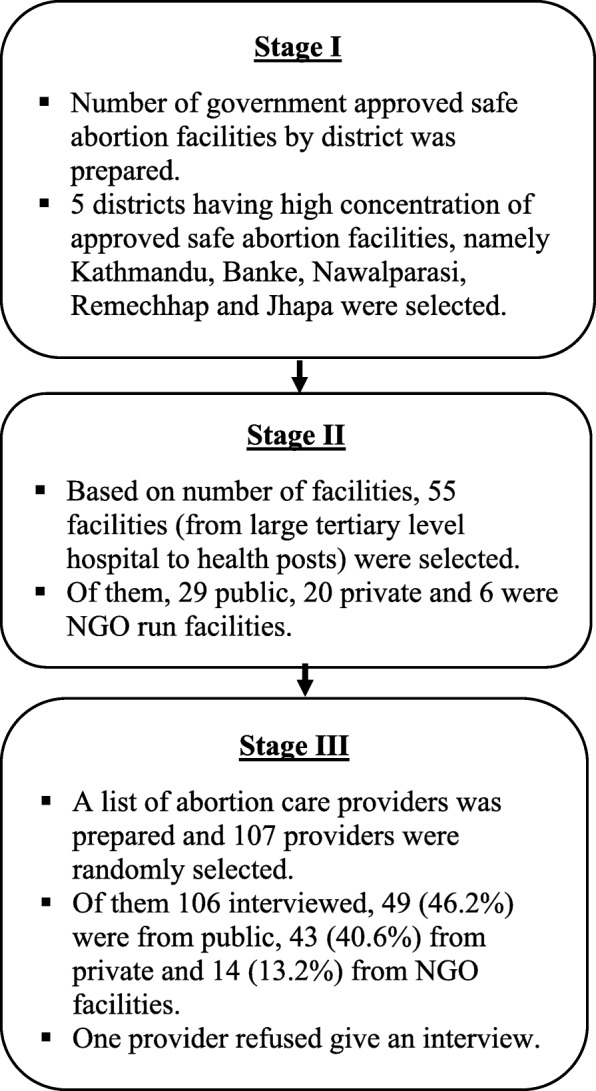
Method of participant’s selection

We conducted a structured interview survey with abortion care providers employed at government-approved facilities that provide abortion care (including government, private, and NGO facilities). Three-stage random sampling procedures were used to select respondents. In the first stage, district-wise number of government-approved safe abortion facilities were listed and five districts having highest number of government-approved safe abortion facilities were selected (Kathmandu, Banke, Nawalparasi, Rammechhap and Jhapa). There were 195 eligible facilities in the five sampled districts. In the second stage, 55 out of 195 facilities (from large tertiary level hospitals to the lowest level health posts) were selected to maximize diversity of facility type (health system level and public vs. private) and type of providers at a given facility. Out of these 55 facilities, 29 were public, 20 private and 6 were NGO-run. The field researchers on this project prepared a list of providers at each facility by contacting hospital and clinic administrators. Among 107 providers we approached, only one provider declined to participate in the interview. In summary, participants were eligible for an interview if they were working in a government-approved safe abortion facility, were directly involved in providing abortion care services (either medical or surgical or both or involved in providing counseling service), and were either physicians, mid-level providers or counselors (Fig. 1).

A structured questionnaire was developed in English and translated into Nepali. The questionnaire was pre-tested with six providers working in abortion facilities not sampled for the study, and was subsequently revised based on pre-test results. Questionnaires took about 45 min to administer and were comprised of six parts: facility background, socio-demographic background, service provision for unintended pregnancy, denial of abortion services, knowledge about legal abortion service provisions, quality of care, and barriers to service provisions. Most of questions were close-ended, although a few open-ended questions were included. We asked providers whether they had ever denied abortion care to patients as well as how often they currently deny care by reason (religious and moral beliefs; high gestational age; contraindications; bulky uterus; lack of skilled service provider, surgical abortion services, or medication; inability to pay service fee; lack of previous children; sex selective abortion or lack of valid reason for abortion). We also asked how many women typically sought abortion services per month and how many were denied abortion service for any reason. Provider attitudes were assessed by using 12 abortion-related value statements (i.e. six positive and six negative attitudinal statements) regarding safe abortion services.

Questionnaires were administered in Nepali by five trained interviewers over a period of 4 weeks in each facility in August 2017. This study was approved by the Nepal Health Research Council. Study participants were fully informed about the study’s research objectives and confidentiality of the data. Written consent was obtained from all participants.

All completed questionnaires were manually reviewed, cleaned, and coded before entering into a computer-based program, Census and Survey Processing System (CSPro) for analysis. A central focus of the analysis was to assess service provider knowledge about the abortion law, their attitudes about abortion, and their experiences denying abortion services. Descriptive analysis was carried out using Statistical Package for Social Sciences (SPSS) version 22. We tested for differences in reasons for denial, referral to a second trimester provider, attitude toward abortion and barriers to providing by provider type (mid-level vs. physician), type of facility (public hospitals, private hospitals and NGO clinics, and primary health care centers and health posts) and whether they have received specific training in abortion care (yes, no) using bivariate analysis.

## Results

### Profile of respondents

In total, 106 providers were interviewed (one of the 107 selected refused to give an interview), including 49 (46%) from public facilities, 43 (41%) from private facilities, and 14 (13%) from NGO facilities (Fig. 1). One-third were obstetricians, gynecologists or other physicians. Two-thirds (66%) were mid-level providers (nurses, auxiliary nurse midwives and counselors). We combine the mid-level providers because we find significant overlap in training between those in nurse, auxiliary nurse midwife and counselor roles; most of those in counselor roles were also trained as nurses or auxiliary nurse midwives. The mean age of respondents was 42 years (range 22 to 69 years) and most were female (82%). Providers had an average of 17 years of health care experience and an average of 9 years of experience at the specific recruitment site (7.3 among physicians and 9.3 among mid-level). Most worked in urban areas (89%). According to providers, an average of 39 women per month seek about services from their facilities (range: 1 to 300). More than a third (39%) of mid-level providers and most physicians (83%) were working either in a private hospital or a NGO clinic. 80% of physicians and mid-level providers reported that they had received formal training on how to provide a safe abortion. More physicians (80%) than mid-level providers (37%) reported that they had received training for management of post-abortion complications (Table 1).

**Table 1:** Profile of sampled respondents

Background characteristics	Physician		Mid-level		Total	
	%	n	%	n	%	n
Current age (in years)						
Mean (SD)	43 (10.7)	36	41 (10.9)	70	42 (10.7)	106
Range (min-max)	26–69	36	22–60	70	22–69	106
Sex						
Male	44	16	4	3	18	19
Female	56	20	96	67	82	87
Years of experience						
< 5	19	7	11.4	8	14.2	15
5–10	17	6	16	11	16	17
More than 10 years	64	23	73	51	70	74
Mean (SD)	16 (10.5)	36	18 (10.6)	70	17 (10.6)	106
Level of education						
Obs/Gyns	61	22	–	–	21	22
Other physicians	39	14			13	14
Mid-level providers (nurses, auxiliary nurse midwives, and counselors)	–	–	100	70	66	70
Location of facility						
Urban	100	36	83	58	89	94
Rural	–	–	17	12	11	12
Types of facility						
Public district and above level hospital	11	4	11	8	11	12
Private hospitals and NGO clinics	83	30	39	27	54	57
Primary health care centers and health posts	6	2	50	35	35	37
Received formal training on safe abortion service (on any methods of abortion)						
Yes	89	32	77	54	81	86
No	11	4	23	16	19	20
Received training on incomplete abortion or pregnancy complications						
Yes	81	29	37	26	52	55
No	19	7	63	44	48	51
Total	100	36	100	70	100	106

### Denial of abortion services

Almost all providers (96%) reported that they had ever refused clients for abortion. Providers report turning away an average of 25% of women seeking services. The most common reasons for abortion denial reported by providers included beyond 12 weeks of gestational age (93%), sex selective abortion (86%), and women’s health/possible contraindication (85%).

One third of providers reported that they sometimes denied women abortions for reasons that have no basis in the law or clinical standards including, for example, the woman does not already have a child, she came repeatedly for abortion services, she was an unaccompanied and unmarried woman or adolescent, or the case was not aligned with the clinician’s personal religious and moral beliefs about abortion. There were no differences in denials for illegitimate reasons by provider type, facility type or training in abortion care.

Over a quarter of providers (28%) denied women abortions for advanced gestation without screening them for indications for legal second trimester abortion, or referring elsewhere, with no differences by facility type or provider type. Providers who received training in safe abortion care were less likely to incorrectly deny women abortions after 12 weeks (23% vs 50% among those with no training).

One quarter of providers (24%) reported denying women abortions due to limited capacity, such as lack of medications or skilled providers. Mid-level providers were much more likely than physicians to report capacity problems (33% vs 6%). Capacity problems were much more common among primary health centers and health posts (54%) than among public hospitals (8%) or private/NGO (7%) (Table 2).

**Table 2:** Denial for abortion services and its reasons

Ever denied abortion service (%)	Physician (*n* = 36)	Mid-level (*n* = 70)	Total (*N* = 106)
Yes	97	98	96
No	3	4	4
If yes, reasons for denial (% of yes only)			
Gestational age above 12 weeks	94	93	93
Sex selective abortion	92	83	86
Maternal health contraindications	83	76	85
Bulky uterus	27	54	45
Gestational age above 8 weeks	19	46	37
We do not provide surgical abortion	6	46	32
Lack of “valid” reason for abortion	31	31	30
Lack of medications	6	33	24
Woman does not have any children	17	27	24
Lack of skilled provider at facility	8	20	16
Woman unable to pay service fee	6	6	6
Providers’ personal religious and moral beliefs about abortion	3	3	3

Only one-third of providers reported that they never deny women for invalid reasons (including based on reasons inconsistent with law or clinical practice, turning women away for advanced gestation without screening and referral, or denying women abortions due to capacity problems). Physicians are more likely than other clinicians to report that they never turn women away for any of these invalid reasons (44% vs 24%) *(Table not shown).*

### Services after denial of abortion services

Although all service providers claimed that they recommend alternatives to women who are denied abortion services, most providers (87%) also stated that they recommend women to continue their pregnancy. Over half of providers (58%) said that they do not have any formal network established for referrals. One in ten providers (10%) said that they refer women to private hospitals for abortion.

Advanced gestational age and possibility of complications were the main two situations in which providers refer women to other facilities for abortion care.

Providers were not sure what women do after they are denied abortion. Many reported that women visit private hospitals for abortion services after denial (78%) or that they continue the pregnancy (67%). Over a quarter of providers (28%) indicated that women visit unsafe/unlisted health providers or buy and consume medicines from pharmacies.

### Knowledge about legal abortion service provision

**Fig. 2:**
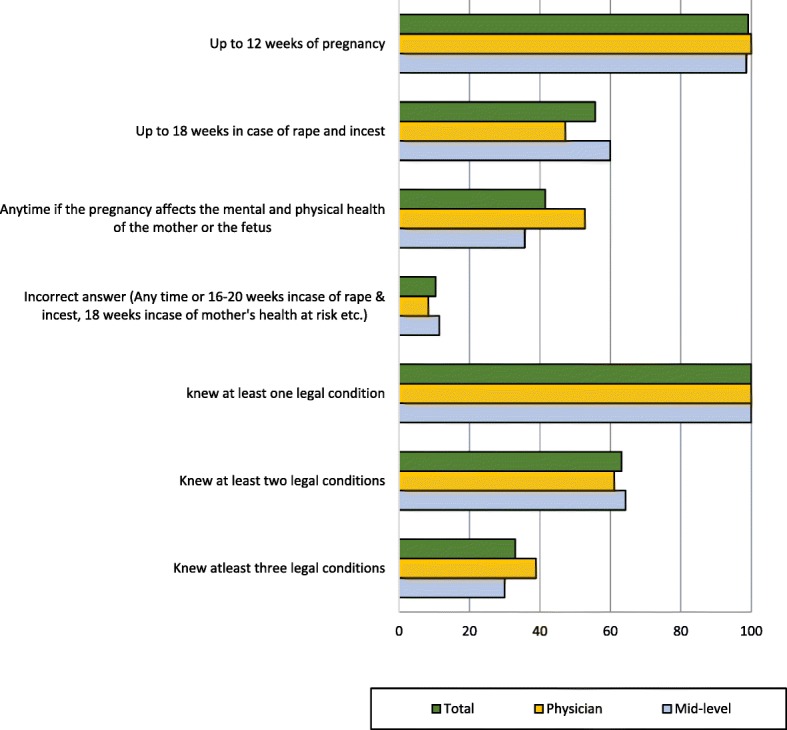
Knowledge about legal provisions of abortion

Though all providers were aware that the law permits women to request abortion care for any reason up to 12 weeks of pregnancy, only 33% of providers knew all three legal conditions under which a woman can seek abortion legally in the country (less than or equal to 12 weeks of pregnancy on request, up to 18 weeks for rape or incest, and any time for maternal or fetal health risk). The least known of the three was the third indication relating to the mental or physical health of the mother or the fetus (42%) (Fig. 2).

Only 9% of providers could spontaneously name three or more of the 11 mental health conditions that make women eligible for abortion after 12 weeks of gestation. After probing, the proportion increased to 43%. No service providers could mention all eleven mental health conditions without probing. A higher proportion of physicians than mid-level providers were aware about the mental health conditions for legal abortion (Table 3). One in five providers (22%) did not know a certified second trimester abortion provider to whom they could refer patients. Providers who received training on safe abortion were more likely than those who had not to know of a place for legal abortion in the second trimester (84% vs. 55%). Neither facility type nor provider type was associated with knowing a second trimester provider.

**Table 3:** Knowledge about mental health conditions for legal abortion after 12 weeks of pregnancy

Mental health conditions	Physicians (*N* = 36)			Mid-level Providers (*N* = 70)			Total (*N* = 106)		
	Yes without probing (%)	Yes with probing (%)	No/DK (%)	Yes without probing (%)	Yes with probing(%)	No/DK (%)	Yes without probing (%)	Yes with probing (%)	No/DK (%)
Difficulty falling asleep	14	31	69	4	29	71	8	29	71
Always sleepy or falls asleep all the time	14	25	75	3	14	86	7	18	82
Lethargic and less energetic	11	25	75	3	11	89	6	16	84
Feels guilty or worthless all the time	6	56	44	1	23	77	3	34	66
Problems concentrating, carefully thinking or making decisions	11	44	56	6	27	73	8	33	67
Excited, restless or irritated	8	39	61	6	20	80	7	26	74
Hesitation participating in recreational activities	3	22	78	–	7	93	1	12	88
Feeling that life has become meaningless and support less	11	56	44	6	29	71	8	38	62
Feeling unable to take care of other child(ren) financially, mentally and physically	28	47	53	10	34	66	16	39	61
Believes baby will affect her education and professional career	6	14	86	–	16	84	2	15	85
Believes pregnancy is result of her extramarital affair	3	33	67	–	33	67	1	33	67
*Knew any three or more mental health conditions (out of 11 listed above)*	*19*	*56*	*44*	*4*	*37*	*63*	*9*	*43*	*57*

### Provider attitudes towards abortion

Overall, service providers were in favor of safe abortion access but a substantial proportion of providers had mixed attitudes or negative attitudes towards certain statements related to safe abortion services. All providers agreed to the statements: “The needs of a patient are more important than the beliefs of a clinician” and “Every woman has the right to access safe abortion to the full extent of the law”. However, one in seven providers agreed with the statement “the later the gestational age, the more sinful the abortion” and one in twenty providers agreed to the statements ‘I feel guilty about providing abortion’ and ‘I feel that providing abortion is morally wrong’. There was no major difference in attitudes toward abortion by type of providers, facility or whether they had received safe abortion training (Table 4).

**Table 4:** Belief and attitudes towards abortion services (% of agree)

Statements	Physician (*n* = 36)	Mid-level (*n* = 70)	Total (*n* = 106)
The needs of a patient are more important than the beliefs of a clinician	100	94	96
Clinicians have a responsibility to counsel patients against having an abortion	3	3	3
Every woman has the right to access safe abortion to the full extent of the law	100	99	99
Providing abortions is a positive contribution to society	89	77	81
I feel that providing abortions is morally wrong	3	4	4
I feel guilty about providing abortions	6	6	6
I do/would worry about telling people that I provide abortions	6	–	2
A woman who has had an abortion brings shame to her family	–	1	1
Women have abortions to take better care of the children they already have	67	83	77
The later the gestational age, the more sinful the abortion	11	16	14
I would continue to be friends with someone if I found out that they had an abortion	92	91	92
Most abortions could be provided under the legal ground of mental health	58	57	58

### Barriers to abortion service provision

Over 60% of providers reported barriers to providing quality abortion services. The most common barriers were irregular supply of medical abortion drugs (25%), lack of trained providers (23%), lack of trained staff (23%), and lack of separate room for providing abortion services (21%). Providers at private hospitals and NGO clinics were less likely to report a lack of these resources than providers at public hospitals, primary health care centers and health posts.

## Discussion

Our study assesses knowledge, attitudes and experiences of providing abortion services among formal abortion care providers in Nepal. This study corroborates previous evidence indicating that many Nepali women face barriers to abortion care and experience unnecessary denial of legal abortion services in spite of Nepal’s liberal abortion law [6]. Abortion care denial is often inconsistent with Nepal’s Safe Abortion Policy [2]. Three types of particularly problematic denials include denials due to limited facility capacity, failure to offer care beyond 12 weeks, and refusal of care for reasons inconsistent with law or clinical practice, such as being nulliparous, young, or unmarried.

Denial of abortion partly due to providers’ lack of full understanding of the legal indications, especially mental health indications, and barriers to providing care, such as a lack of trained personnel and supplies. To a lesser extent, denial of care also results from practices of refusing services for reasons that are not based on the law or quality clinical care but may be driven by provider judgment of women, or moral and religious beliefs. Though most providers claimed that they refer women to other facilities after denying them abortion, about half lack formal referral networks to do so. Moreover, most providers stated that they recommend that women turned away carry their pregnancy to term. These findings are consistent with previous studies focused on women’s experiences being denied legal abortion services in Nepal [6, 7]. Roughly a quarter of providers reported suspicion that their patients may attempt self-induced abortion outside of the formal health system following denial. This is also consistent with findings that show the majority of women presenting to hospitals in Nepal with complications following induced abortion of pregnancy had undergone medically induced abortions using unknown substances acquired from uncertified sources [10].

These results come from five districts with the highest volume of abortion provision in the country; it is possible that access to abortion is poorer and barriers to care are greater in the districts we did not survey. In addition, the survey questions used for this study ask specifically about individual provider experiences with denial of abortion and knowledge of the law; it is important to acknowledge however that all participants surveyed in this study do not have equal decision-making power when it comes to whether or not a patient will receive care. Typically, a health risk evaluation is completed and supervised by either a physician or head-nurse; therefore, not all participants (in particular nurses and counselors) were necessarily the ones who made the final decision. The data presented are not able to differentiate between abortion denials made by an individual and denials made by the clinic. Provider responses to the study survey are also subject to recall bias, which may affect providers with more years of experience disproportionately.

## Conclusions

Though significant progress has been made in expanding abortion service provision in Nepal, ensuring that all women seeking to terminate a pregnancy receive legal and safe abortion care remains an important challenge. Many providers highlighted very important both supply-side barriers to providing quality abortion services (such as low knowledge about legal abortion access and barriers to service provision). Shortages in trained providers, supply of medical abortion drugs, and facility space were the major barriers to supply, which prevent many women from accessing legal abortion services within the first 12 weeks of pregnancy, when abortion should legally be available on request in Nepal. Given poor knowledge about the abortion laws and service provision indications among abortion care providers, provider training (or refresher training for those who are already trained) at all levels of the health care system are necessary to ensure the availability of quality abortion care. Emphasis must be on increasing providers’ capacity to assess medical and legal eligibility for abortion services, including knowledge of mental health conditions for second trimester abortion, and to provide counseling for women and referral as needed. Additional investigation of provider knowledge through qualitative interviews and analysis could provide further insight in to the reasons behind gaps in provider knowledge and preferred strategies for improving provider training and knowledge of abortion care. Since some providers appear to deny abortion care due to personal and moral beliefs, training for providers might also include value clarifications so that providers can practice what they profess to believe: “The needs of a patient are more important than the beliefs of a clinician” and “Every woman has the right to access safe abortion to the full extent of the law.”

## Abbreviations

CSPro: Census and Survey Processing System
MVA: Manual Vacuum Aspiration
NGO: Non-Government Organizations
SPSS: Statistical Package for the Social Sciences
